# High Throughput Profiling of Molecular Shapes in Crystals

**DOI:** 10.1038/srep22204

**Published:** 2016-02-24

**Authors:** Peter R. Spackman, Sajesh P. Thomas, Dylan Jayatilaka

**Affiliations:** 1 University of Western Australia, Dept. of Chemistry, Crawley, Western Australia, 6008, Australia

## Abstract

Molecular shape is important in both crystallisation and supramolecular assembly, yet its role is not completely understood. We present a computationally efficient scheme to describe and classify the molecular shapes in crystals. The method involves rotation invariant description of Hirshfeld surfaces in terms of of spherical harmonic functions. Hirshfeld surfaces represent the boundaries of a molecule in the crystalline environment, and are widely used to visualise and interpret crystalline interactions. The spherical harmonic description of molecular shapes are compared and classified by means of principal component analysis and cluster analysis. When applied to a series of metals, the method results in a clear classification based on their lattice type. When applied to around 300 crystal structures comprising of series of substituted benzenes, naphthalenes and phenylbenzamide it shows the capacity to classify structures based on chemical scaffolds, chemical isosterism, and conformational similarity. The computational efficiency of the method is demonstrated with an application to over 14 thousand crystal structures. High throughput screening of molecular shapes and interaction surfaces in the Cambridge Structural Database (CSD) using this method has direct applications in drug discovery, supramolecular chemistry and materials design.

Molecular shape plays a fundamental role in our understanding of chemistry and biochemistry, with the supramolecular assembly of molecular species generally being understood both in terms of the intermolecular interactions and the complementarity of molecular shapes. In this area, Lehn’s conception of supramolecular chemistry focused on specific molecular recognition leading to ‘supramolecules’^1^. Similarly, much of the known molecular recognition processes (such as drug-receptor binding, enzymatic reactions etc.) can be understood with the ‘lock and key’ paradigm of steric fit proposed by Fischer^2^ over a century ago.

Much of our present understanding of crystal structures, from Kitaigorodskii’s principles of crystal packing^3^–the maximum utilisation of space and minimisation of free energy—to Desiraju’s approach to crystal engineering^4^–the notion of ‘supramolecular synthons’ as recognition units based on chemical functionality—has emphasised the importance of molecular shape.

Yet, beyond cartoon depictions of shape complementarity and qualitative arguments the role of molecular shape is largely sidelined in quantitative analysis of supramolecular chemistry and crystal engineering. It is our view that this is largely due to the lack of a straightforward method allowing us to incorporate molecular shape into such studies. It would appear, then, that computational approaches for describing molecular shapes in an accurate, systematic manner are highly desirable.

With regard to molecular crystals, it is hard to overstate the scientific value that has been derived from the magnitude of experimental crystal structure data available, curated within the Cambridge Structural Database^5^. Such a database represents an opportune target for the purposes of quantitative analysis. To adequately utilise this growing amount of data, automation and algorithmic analysis (be it traditional statistical methods or machine learning) are required. So, for the dual purposes of classification and understanding molecular shapes in crystal structures, we present an efficient computational method based on spherical harmonics with the capacity to accurately describe molecular shapes in their crystal environment.

In order to account for molecular shape, some degree of chemical information (i.e. the effects of different elements), and some aspects of the crystal environment, we utilise the Hirshfeld surface^6^ (HS) as a summary object of molecules in crystals. The HS developed by Spackman *et al.* is an isosurface surrounding a molecule in its crystal structure, defined as the boundary where the contribution of electron density ‘belonging’ to the molecule is equal to that of its crystalline surroundings. The densities in each case are approximated by a superposition of quantum mechanically derived spherical atomic densities, a so-called promolecular^7^ density. Much like van der Waals or CPK^8^ surfaces, the HS represents a realistic molecular surface, albeit one generated from the experimental X-ray geometry. Further, it encompasses information about the packing and intermolecular interactions within a crystal, and includes some molecular size effects, both of which may be encompassed in its shape.

The HS is often decorated with properties such as *d*_i_ (the distance to the nearest internal atom), *d*_e_ (the distance to the nearest external atom), and *d*_norm_ (the distance between internal and external atoms normalised by van der Waals radii). Figure 1 demonstrates some of the capacity for these properties to visually represent aspects of the crystal environment, such as the red spots here indicating close contacts between molecules.

HS properties, namely *d*_e_ and *d*_i_, have been previously used by Gilmore and co-workers^9^,^10^ when they proposed so-called ‘genetic fingerprinting’ based on rasterisation of the Hirshfeld fingerprints^11^,^12^ (2D histogram representations of *d*_e_ and *d*_i_ from the HS). This constituted a similar use of the HS as a summary object of molecules in crystals, but the descriptors used by these workers do not directly express the shape of the HS. As such they do not provide a straightforward means of incorporating molecular shape in quantitative analysis.

Outside of the domain of molecules in crystals, there are a number of other methods routinely applied to surfaces (e.g. Van der Waals surfaces) and domains (e.g. binding pockets in proteins) that represent shapes, molecular or otherwise, using spherical harmonic functions. Rotation invariant descriptors have been been applied in the field of drug design^13^,^14^ and more generally in 3D shape recognition^15^,^16^, where they are used to perform shape matching without the high computational cost of ‘docking’. Docking essentially consists of rotating the two shapes prior to comparison so that they are in maximum coincidence, and it rapidly becomes an extremely costly step in the comparison procedure as large numbers of structure comparisons are required.

An important aspect of utilising spherical harmonic descriptors is the natural parameter *l*_max_ (i.e. the highest order of spherical harmonic functions used in the transform) which provides a systematic parameter of the level of detail in the shape description. As a brief example of of how accurate the description may be for higher values of *l*_max_, Fig. 2 shows two HS reconstructions (i.e. meshes generated from the resulting coefficients of the spherical harmonic transform), one for the benzene crystal and the other for an indomethacin crystal. Both reconstructions include the *d*_norm_ property which colours the surface. In practice we have found that typically 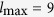 constitutes an acceptable compromise between precision and brevity in the description of the HS.

Since the method outlined here also involves rotationally invariant shape descriptors, it enables computationally efficient shape matching in a very large number of structures (taken here from the CSD). The full description of the details of the method, along with a brief description of the HS, is provided in the methods section.

The potential value of an efficient, numerical, rotation invariant description of the HS in a crystal will be demonstrated here through its application to selected sets of crystal structures. The first dataset consists of 29 metallic crystal structures; a mix of hexagonal close-packed (HCP) and cubic close-packed (CCP) crystal lattices. The second set consists of over 300 structures; comprising a series of substituted benzenes, naphthalenes and phenylbenzamides, along with some pyridine analogues of each kind. The separation and grouping both these datasets in a principal component analysis (PCA) and cluster analysis based on the shape descriptors is discussed with reference to crystal packing, chemical scaffolds, chemical isosterism and molecular conformation. Finally, the computational efficiency of this technique will be outlined by examining a dataset comprising over 14,000 organic crystal structures.

## Results and Discussion

### Hirshfeld surfaces of metallic crystals

A relatively simple case of the association of the HS with the packing of a crystal may be found in metallic crystal structures. As such, examining a small set of metallic crystal structures constitutes an ideal first step toward demonstrating the capacity of this technique to adequately describe HS shape. The full list of metallic crystal structures may be found in Supplementary Table S1 online.

The first dataset may be classified into two distinct categories: cubic close packed (CCP) and hexagonal close packed (HCP) structures. These two categories have HS with distinct shapes corresponding to their packing, as the HS is dependent only on the electron density (and, by extension, the interatomic distances and symmetry in the crystal lattice) of the individual atoms; the surface directly represents their packing environment. A successful description technique will provide the capacity to separate these two categories algorithmically, whether by clustering algorithms or visually partitioning the space using plots projected onto the PCA axes.

**Figure 3:**
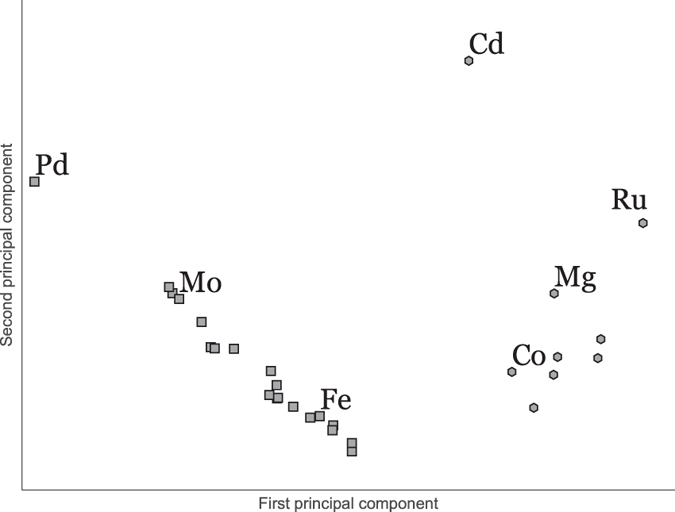
2D PCA for the metallic crystals dataset, with squares indicating CCP structures, and hexagons indicating HCP structures.

**Figure 4:**
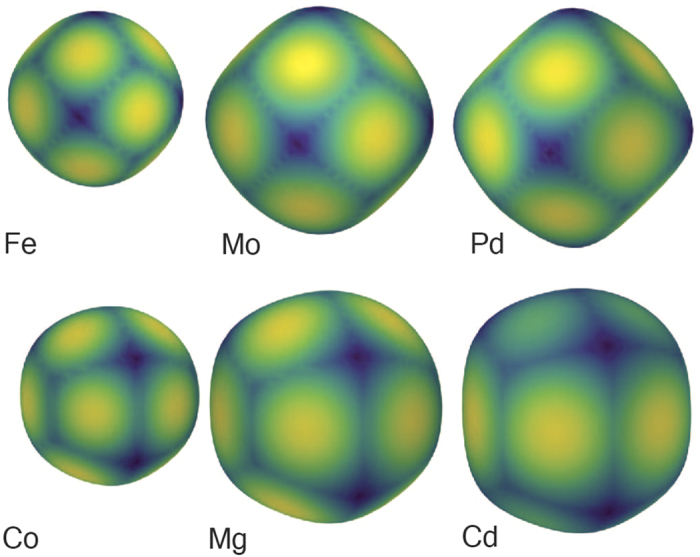
Hirshfeld surfaces for 3 CCP metals and 3 HCP metals with *d*_norm_ mapped on the surface. Note the distinct patterns in both the shape of the surface and *d*_norm_ correspond to the lattice structure in the crystal environment, along with the heightened tendency toward asphericity as the packing becomes ‘tighter’ (closer interatomic distance with regard to electron density).

Figure 3 shows a scatter plot with axes of the two first principal components of the feature space. The PCA was performed on feature vectors consisting of first 10 rotation-invariant shape descriptors of each HS shape. Clearly, the objects in the descriptor space separate into two categories. One of the groups (CCP) is almost linear in the 2D PCA, indicating one dimension of variation within the group (i.e. unit cell size with respect to atomic radius). The proximity and linearity of CCP metals in the descriptor space may be readily understood in terms of the symmetry constraints brought by CCP: there is no degree of freedom in the shape of the unit cell. Thus the only variation in this group must stem from a variation in interatomic distance and electron density. Indeed, as we traverse the CCP metals along the approximate line from Fe through to Pd (see Fig. 4, we may see the increased similarity between the HS and the space filling Voronoi or Wigner-Seitz cell^17^.

The HCP group, on the other hand, exhibits more variation in its HS shape. This variation may be accounted for by the varying degrees of of anisotropy in the different HCP metals, i.e. the extra degree of freedom in the *c* axis of the unit cell. Examining the ratios of unit cell lengths 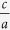 in the HCP metals, it is evident that those with radically different ratios tend to be separated, and the apparent outlier Cd can be explained by its notably large ratio (1.89) i.e. its high degree of anisotropy. Indeed, Cd is the only element with a ratio higher than the ideal of 1.633^18^. The immediate separation of Cd indicates that its unusual degree of anisotropy in the unit cell is directly associated with the HS shape, and that this shape is adequately described by this technique.

Given the sharp differentiation seen for Cd, one might expect, then, that structures with identical unit cell ratio would be co-located in the descriptor space. This is not the case, as while they may have the same symmetry the different atomic lattices may, just as the CCP metals, vary in their electron densities and interatomic distances. For example, while Co and Mg share a close to ideal 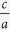 ratio (both have a value 1.62 vs. the ideal value of 1.633) they differ in their interatomic distance within the lattice, with Co being rather more tightly packed (interatomic distance roughly 2.5 Å) than Mg (interatomic distance of 3.2 Å). Thus, unless this discrepancy in interatomic distance is compensated for by a complementary change in the electron density, the two metallic atoms in their crystal environment will have differing HS shapes. This difference may be visualised through the increased asphericity in from Co to Mg in Fig. 4.

The HS shape of simple metallic structures being related to the anisotropy of the unit cell and the crystal lattice itself is unsurprising. Nonetheless, it is informative in that the relationships are also revealed through analysis of the shape descriptors themselves—even when projected only onto two dimensions (principal components) No doubt similar quantitative analysis could be performed through exploring unit cell ratios 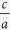, interatomic distances, and electron density parameters, but it is clear that these shape descriptors have the capacity to encapsulate this kind of information—with only minimal direct parameterisation. It is this capacity to store information about both molecular shape and the crystal lattice in which it is embedded that holds immense promise for the application of such methods crystal structures.

### Hirshfeld surfaces of molecular crystals

**Figure 5:**
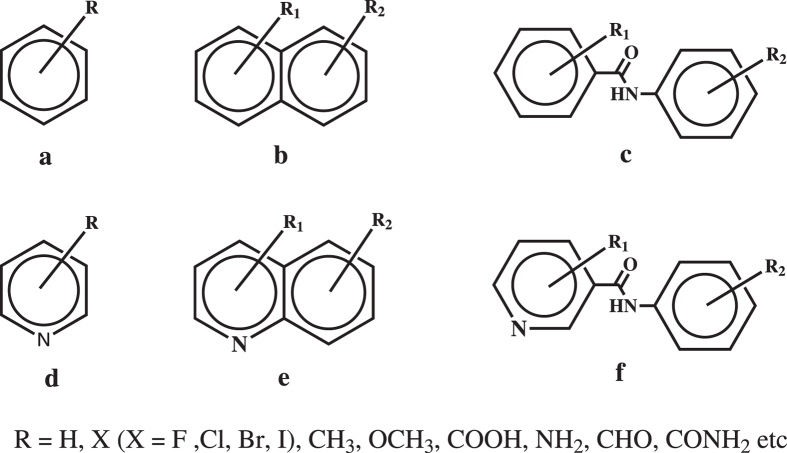
Molecular structures of the three classes of substituted benzenes, naphthalenes and phenylbenzamides and their pyridine analogues examined in this study. Note that the pyridine ring N atom and R group may have varying positions.

We shall now examine a constructed dataset of 309 crystal structures comprising 3 kinds of scaffolds (pictured in Fig. 5): 232 phenylbenzamide type scaffolds (with 21 pyridine analogues), 12 benzene type scaffolds (with 7 pyridine analogues), and 27 naphthalene type scaffolds (with 9 pyridine analogues). All structures have 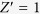 i.e. one molecule in the unit cell. The entire list of 309 molecular crystals and their CSD codes may be found in the Supplementary Tables S2–S7 online.

When examining this larger dataset, of particular interest is not only the capacity of this method to describe more complicated shapes, but its potential to classify crystal structures associated with known scaffold types or other relevant chemical or geometric properties.

**Figure 6:**
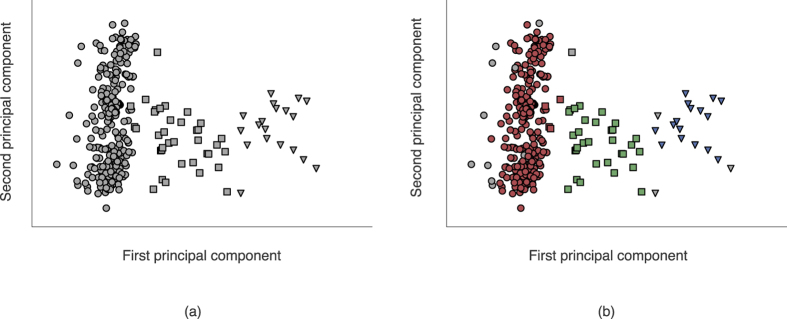
(**a**) 2D PCA projection of selected benzene, naphthalene and phenylbenzamide scaffolds, and (**b**) the same projection coloured by clusters assigned using HDBSCAN. In both plots, circles are used to indicate phenylbenzamide type scaffolds, squares to indicate naphthalene type scaffolds, and triangles used to indicate benzene type scaffolds.

The HS of each structure was described up to 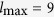 (see Equation 2), and the combination of these shape descriptors and the mean radius constituted the feature vector for use in cluster analysis and PCA i.e. a shape-plus-size descriptor comprising 11 elements. The results may be seen in Fig. 6, and there are 3 broad chemical concepts which are explored in the analysis of the clustering.

#### Chemical scaffold types

We observe that there are some incorrectly assigned objects and some unassigned objects; however, this is quite typical when using clustering algorithms on real world data. The classification of objects will vary depending on the clustering algorithm being used (here we have used HDBSCAN^19^ which is not parameterised by the number of clusters, so in a sense it ‘discovers’ that there are 3 clusters). Still, Fig. 6 demonstrates a clear tendency toward grouping into the three major scaffold categories, corresponding with our prior knowledge of the dataset. The capacity of this technique to provide so clean a separation under PCA (i.e. the tendency of the different classes in this example to occupy distinct regions of the scatter plot) is also promising for further studies with cluster analysis or machine learning.

#### Chemical isosterism and the pyridine analogues

The assignment of three classes using HDBSCAN^19^ on this dataset, and the co-location of the pyridine analogues with their respective base classes accurately reflects our intuition regarding which object belongs in which class. In this manner it can be said to account for some extent of chemical isosterism in the crystal context. Since chemical isosterism is an important concept in the field of drug discovery^20^, this capacity may prove to be of value in future applications.

#### Molecular conformation

**Figure 7:**
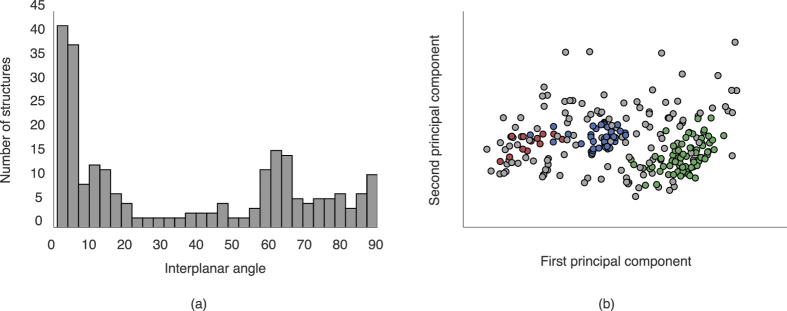
(**a**) A histogram of the interplanar angles between the two phenyl rings in the phenylbenzamides. Note the distinct peaks around 0–20° and 60°, with a more diffuse region between 60° and 90°, and (**b**) A 2D PCA plot of the set of phenylbenzamides alone, again coloured by the clusters from HDBSCAN.

Within the group of phenylbenzamides and their pyridine analogues, there emerge at least two clusters—regions of the dataspace with higher density than their surroundings. This indicates some level of systematic variation within the class with regards to the HS shape of each object. The most obvious explanation for this distribution lies in variation of the interplanar angle (i.e. the angle between the planes made by the phenyl or pyridine rings), which will be associated with different HS shapes in the crystal environment. To confirm this intuition, we may look at the distribution of these interplanar angles in Fig. 7. It is quite clear that the groupings correspond almost directly to those within the phenylbenzamides.

This correspondence holds if we examine the clustering of this group alone i.e. perform the same analysis on the phenylbenzamides alone. While containing more unassigned objects, this set contains two strong clusters, the first of which is associated with the 0–20° peak in the interplanar angle histogram, and the second of which is associated with the 60° peak. The third cluster is much more diffuse, and this is reflected in the more uniform distribution between 70° and 90° in the histogram. The strong association between the distribution of interplanar angles and the distribution of the shape descriptors shows the capacity for this technique to automatically ‘discover’ chemically relevant properties associated with the HS shape, and that these groupings are (in this case) associated with physically meaningful differences in a given dataset.

### High throughput processing of structures in CSD

**Figure 8:**
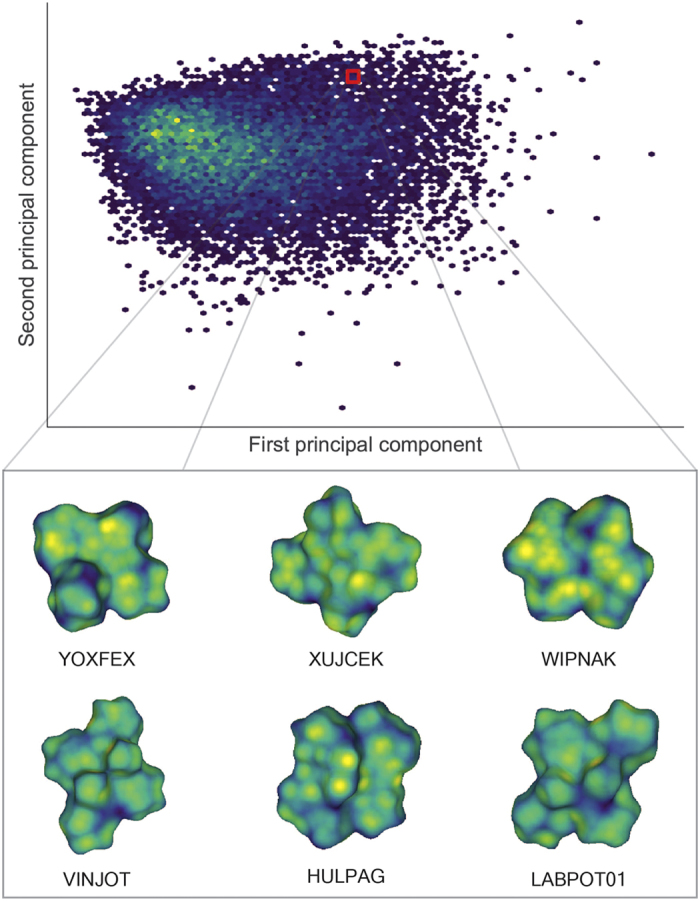
Hexagonally binned 2D histogram of the first two principal components from invariants up to *l*_max_ = 9 along with the mean radius of all 14,772 structures. Note that the the region highlighted as red square in the histogram represents closely related structures in 10-dimensional principal component space, and not necessarily the components in the 2D PCA. The similarity in corresponding molecular shapes can be visualised in the representative structures contained within this cluster.

In order to become a practical tool for clustering or classifying based on common features in large datasets, any method must provide an acceptable degree of speed and efficiency. With this in mind, we have provided the results of this analysis on a larger dataset here. Figure 8 represents the 2D PCA projection of 14,772 non-disordered, 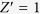 single-crystal structures in the CSD with structure refinement *R*-factor 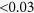, only one molecular residue, the heaviest permitted element was Xe, and for which the HS was ‘star shaped’ (17,646 were not ‘star shaped’).

The meaning of the 2D PCA distribution is not itself the target of discussion (though the lack of clear separation may indicate that two dimensions is not enough to visualise such a diverse dataset). Rather, we emphasise that clustering this dataset takes less than five seconds on a single-processor laptop. This demonstrates the potential for the technique in analysing even larger scale datasets. More details of the speed and efficiency are outlined in the Efficiency and implementation considerations section.

Even though it is not the main focus of this example, it would be remiss not to discuss some aspects of the results obtained from this large cluster analysis. First, we observe very many different small clusters, many of which are nearly identical. These nearly identical clusters usually correspond to several determinations of the same crystal structure (for any reasonable technique it should be expected that duplicate crystal structures will be co-located in the descriptor space). Focusing on one cluster located in the selected region in in Fig. 8, we may see a visual similarity in the HS of the selected objects. This displays the potential of this method to screen for a particular molecular shape or ‘interaction surface’ in the CSD, irrespective of the similarity in chemical structure or elemental composition of the molecules. In other words, this method provides a shape based structural comparison tool which is fundamentally distinct from the conventional chemical connectivity-based CSD search tools^21^.

### Future research and prospects

In this paper we have presented the first application of rotationally invariant spherical harmonic shape descriptors based on Hirshfeld surfaces for analysing the nature of molecular packing in crystals. The advantage of using the technique is that once the descriptors are defined it can be applied without bias, automatically and efficiently on potentially large datasets—too large for direct examination by an individual.

The technique we have developed need not be applied to Hirshfeld surfaces: any surface which is characteristic of the molecular packing in crystals may be used. As outlined, it may also be applied to any properties mapped on the HS, such as 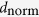 (a property which generally reflects the intermolecular interactions of the molecule).

Further, the capacity to process large-scale datasets provides promise in the fields of drug discovery and crystal engineering. In addition to the conventional drug discovery techniques that largely rely on functionalisation and systematic modification of selected chemical scaffolds, a systematic and quantitative method based on shape affords new possibilities. For example, this technique could be used to profile the shape of protein receptor pockets, along with a property mapped onto the surface of the pocket (e.g. 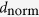 or electrostatic potential), subsequently searching through the large number of diverse structures in CSD extracting the best-matching candidates, on the assumption that the HS of a molecule in a or co-crystal of a molecule constitutes an acceptable proxy for the receptor pocket. Similarly, in the field of crystal engineering and supramolecular chemistry, the molecular shape based approach could help in developing systematic design strategies that utilis e more of the chemical information inherent in shape and interaction surfaces, information that may be difficult to incorporate for a human investigator.

## Methods

### Representing Hirshfeld surfaces with spherical harmonics

The Hirshfeld surface has been used primarily as a graphical object for visualisation. It is an isosurface of a particular function of the type and positions of a subset of the atomic nuclei in an infinite periodic crystal. It is represented as a set of vertices 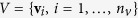 which are connected in triangular facets. Typically, *V* comprises thousands of vertices, making it prohibitively large for search and comparison algorithms on large datasets.

The first step in representing the HS with spherical harmonics is the determination a suitable origin. Since the number of vertices is large and evenly distributed, the mean position 
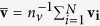
 represents an adequate centre.

Next, the surface must be normalised to have roughly unit radius via the transformation 
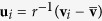
. The mean radius is 
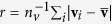
. If **u** is one of the vertices 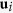, this defines a function on the unit sphere 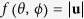 where the polar angles are defined in the usual way by


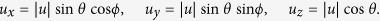


*f* is the normalised HS; it can be defined at points other than the given vertices by interpolation. Note that we consider only surfaces for which there is a unique normalised vertex for every polar coordinate. This restricts the surfaces to so-called star-shaped domains, which comprise the majority of small-molecule HS.

Any function of polar angles such as *f* may be represented to arbitrary accuracy (which may be parameterised by 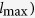 using the spherical harmonic functions, 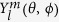, as a basis as follows:


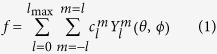


The coefficients 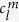 are the spherical harmonic expansion coefficients.


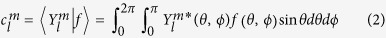


These coefficients 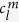 may be more readily computed through the use of a Lebedev quadrature^22^,^23^,


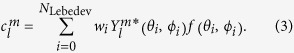


The quadrature weights and points 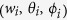 are fixed for a given choice of 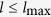. Such grids are widely used in quantum chemistry, and provide an efficient means to exactly integrate polynomials or spherical harmonics on the surface of a sphere. If the summation in (2) is restricted to 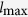 there will be 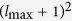 expansion coefficients.

The expansion procedure described above may also be used to encode other properties which are recorded on the same set of vertices in *V*. The procedure is identical except that one uses the set of property values 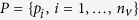 instead of *V* to define the normalised HS i.e. 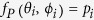. One then obtains the spherical expansion coefficients for this colouring of the surface.

### Rotation invariant shape descriptors

Because we wish to obtain numerical descriptors independent of the orientation of the HS it is desirable to process the coefficients of the spherical harmonic transform such that they are rotation invariant. Weyl^24^ has described the general procedure for constructing all such invariants (see also Biedenharn and Louck^25^). Burel and Henocq^26^ have proposed a more limited set of invariants, and our experience has shown that using only the simplest “**N**” type invariants yields acceptable results. These invariants may be evaluated as follows:


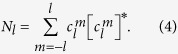


If it is desirable to factor size into the shape analysis, we need only include the mean radius as an additional invariant by appending it to our feature vector for comparison. As previously mentioned, These descriptors may also be applied to quantities decorating the HS or indeed any other scalar function on the HS.

### Efficiency and computer implementation considerations

On average, for the data set comprising 14,772 structures, the HS calculation and analysis took between 1–3 s per crystal structure on a typical Intel single processor laptop. The majority of this calculation time is spent in calculating the triangulated HS. There is potential for great speed up by not triangulating the Hirshfeld surfaces at all, but by calculating the required HS points and the quadrature points directly. For 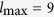 which is more than sufficient for descriptor purposes there are only 50 grid points; this is 2–3 orders of magnitude smaller than the number of points needed for high quality graphical display. This has not been pursued as the software is currently in a proof-of-concept state, and the meshes for the Hirshfeld surfaces themselves are useful for comparison.

All associated surface data has been stored using Hierarchical Data Format HDF5^27^. This has minimal impact on the descriptors themselves (as we have only 10 per surface). Even for 1 million structures the storage of their entire feature vectors up to 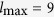 would require less than 100 MB of storage. Thus, the data retrieval aspect of the process requires only a trivial amount of time.

By contrast, the distance calculations required for cluster analysis necessarily scale as 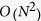 where *N* is the number of structures considered, since we must calculate distances between each possible pair of structures. Therefore, the computation of this distance matrix will become the bottleneck as *N* gets large. Despite this, even for the large data set (14,772 objects) considered here, the total computation for HDBSCAN clustering (once the shape descriptors have been calculated) was less than five seconds on a consumer-grade laptop.

It is the efficiency of the representation of shape here that will allow shape to be incorporated into further algorithmic analysis of the CSD (or any other crystal structure databases). Such possibilities will be explored in future publications.

## Additional Information

**How to cite this article**: Spackman, P. R. *et al.* High Throughput Profiling of Molecular Shapes in Crystals. *Sci. Rep.* **6**, 22204; doi: 10.1038/srep22204 (2016).

## Supplementary Material

Supplementary Information

## Figures and Tables

**Figure 1:**
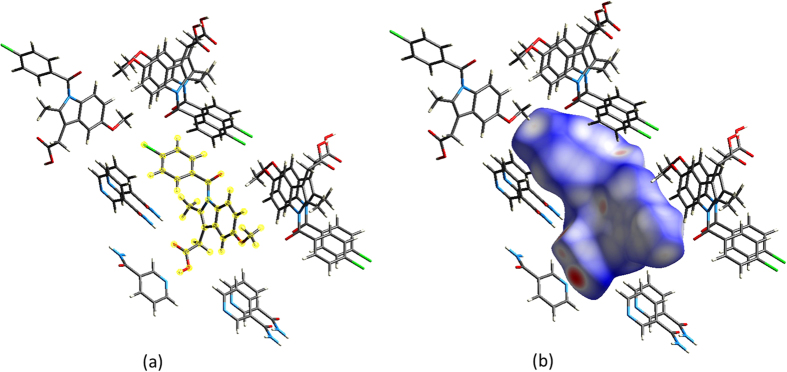
Views of (**a**) crystalline environment in the co-crystal of the anti-inflammatory drug indomethacin with nicotinamide, and (**b**) corresponding Hirshfeld 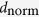 surface around indomethacin. Close contacts appear as red regions, while more distant interactions will appear from white to blue.

**Figure 2:**
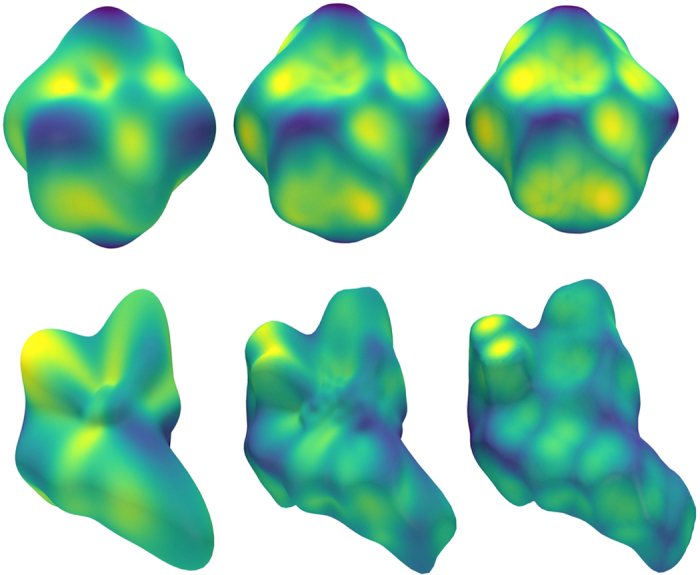
From left to right, reconstructed (*l*_*max*_ = 9 and *l*_*max*_ = 20) and original Hirshfeld surfaces for BENZEN07 (top) and INDMET (bottom). Surfaces have been coloured based on the *d*_*norm*_ property at each vertex. While the reproductions at *l*_*max*_ = 9 are not exact, descriptions at this level clearly capture the essential idea of the shape of the HS.
