# Supplementary material for: High Throughput Profiling of Molecular Shapes in Crystals
Source: Sci Rep. 2016 Feb 24;6:22204. doi: 10.1038/srep22204 (PMC4764928; doi:10.1038/srep22204)
Supplement: Supplementary Information [file srep22204-s1.pdf]

High throughput profiling of molecular shapes in crystals: Supplementary Information

Peter R. Spackman[1,*], Sajesh P. Thomas[1], and Dylan Jaytilaka[1]

[1]University of Western Australia, Dept. of Chemistry, Crawley, Western Australia, 6008, Australia
[*]spackp01@student.uwa.edu.au

Data and tables

All CIF files were retrieved from the Inorganic Crystal Structure Database (ICSD) in the case of metallic crystals, or Cambridge Structural Database (CSD) in the case of molecular crystals.

Supplementary Table S1. Metallic crystal structures used

Element	ICSD Code	Crystal System
Ag	64706	CCP
Ba	56132	CCP
Be	1425	HCP
Ca	53768	CCP
Cd	619639	HCP
Co	52935	HCP
Cr	41505	CCP
Cs	44754	CCP
Fe	41506	CCP
K	44756	CCP
Li	44759	CCP
Mg	52260	HCP
Mn	41509	CCP
Mo	41513	CCP
Na	44757	CCP
Nb	41512	CCP
Ni	41508	CCP
Pd	41517	CCP
Rb	44755	CCP
Rh	41516	CCP
Ru	40354	HCP
Sc	164088	HCP
Sc	41502	CCP
Sr	44721	CCP
Tc	41514	CCP
Ti	41503	CCP
Ti	43416	HCP
Y	660002	HCP
Zr	53785	HCP

Supplementary Table S2. Phenylbenzamide CSD codes

ACAKEU	FITLUR	IWAQON	MAZWIU	QITTOD	WAFNEX
ACAKOE	FOWXIZ	IWAQUT	MIGVAA	QITTUJ	WAWMAJ
ACALAR	GAQGIQ	IWARAA	MISCEX	QITVAR	WAWMEN
ACALEV	GEHKIN	IWAREE	MODSOO	QIXMUG	WIGYOC
ACALIZ	GISYOX	IWARII	MUBBUH	QUKVUN	WIPFUX
ACALUL	GIXDOI	IWAROO	MUBHEX	QUYGAT	WOJLIS
ASAYID	GOLWAG	IWARUU	NAQSUT	RIYQIA	WOMWUR
AWEWAB	HEBFUR	IWASAB	NICHOY	ROFMEF	XIYXOT
AXAHUD	HEXXUE	IWASEF	NIDGAK	ROGHAX01	YAGBIT
AYOTOY	HIGCAB	IWASIJ	NORHUY	SABFIM	YAYCEI
AZALOD	HIRGOF	IWASOP	NUQGAI	SAFRUO	YAYJUF
AZUVOH	HIRROR01	IWASUV	NUQLIV	SAFVIG	YEGJID
BATYAY	HISDIX	IWATAC	NUQWEC	SAGLOD	YEGJID01
BEBMEB	HIVCOF	IWATEG	OBOXEK	SAGYUW	YEGJOU
BUTDID	HIZVUJ	JABWIT	OBUCEV	SAMBOY	YEGJUP
BUTDOJ	HOLKOJ	JAVWIN	OBUWOZ	SAMBUE	YIQWAX
BZANIL02	HOZCEF	JAVWIN01	OCAGOQ	SAMCAL	YIQYED
CEGVOB	HUDXEK	JIZRUH	OCARER	SAMCAL01	YIRHOX
COQSAD	HUGLAX	JODTEB	OCEMIU	SAMCEP	YIRHOX01
CUZZUS	HULHAX	KADZEU	OJUJEJ	SAQKEA	YOCVES
DIBDAU	ICLSAL	KEMREZ	PADTIX	SAQKEA01	YODDUR
DIBDEY	ICULEX	KIZRIV	PADTUJ	SECDEL	YODFAZ01
DIBDIC	ICULOH	KODTUT	PAVMAC	TAQBIX	YODMOU
DIBDOI	IKAYOJ	KODVOP	PAXNEJ	TEGLEW	YOTROP
DIBDUO	IMARUK	KOFDOZ	PCHSAN	TIGMEC	YOVNED
DIBFAW	INODIY	KUVJER	PCHSAN01	UCOVAJ	YUQLIG
DIBFEA	INODUK01	LAPHAN	PIFLAS	VABVEB	YUQMAZ
DIBFOK	IQOHIF	LAPZAF	PILMIH	VACCOT	YUXMEK
DIMTOK	IWAMOJ	LAQDOY	POPVUM	VECCAH	YUYTOB
DIWRAD	IWAMUP	LASHIY	POPWUN	VECCEL	ZAPWOE
DIXDOD	IWANIE	LASHOE	PUFFEC	VIKHIH	ZAPXUL
DUSDAX	IWANOK	LASJAS	PUFLEI	VIPGOR	ZOQWUZ
EFAZES	IWANUQ	LEFKEO	PUGKUY	VOFBID	
ENUKAA	IWAPAY	LEPNEA	QALDEO	VOLJIR01	
EVAHUF	IWAPEC	LEPNIE	QEDLES	VOLJUD	
EVIXOX	IWAPIG	LEPNUQ	QITSOC	VOQJAN	
FABBAN	IWAPOM	LEPPAY	QITSUI	WACQAU	
FACYAK	IWAQAZ	LESLIG	QITTAP	WADBEK	
FENPIX01	IWAQED	LIVZOG	QITTET	WADCEL	
FICLAG	IWAQIH	MAJROG	QITTIX	WAFKAR	

Supplementary Table S3. Phenylbenzamide pyridine analogue CSD codes

AMUCUH
AMUDIW
AMUFAQ
COFVOK
DIFMOW
FOBMEP
HIFWUO
NOPNIR
PEDDIM01
PELKUN
PELKUN01
PELLOI
PELLUO
PYDCXA10
PYDCXA11
QEKNUQ
TICDOZ
TICDOZ01
UXEXOL01
UXEYAY
ZAQNIQ

Supplementary Table S4. Benzene CSD codes

ABUMIT
AMPHOL01
AMPHOM03
BENZEN07
BZONTR
DCLBEN03
FACFAQ
FACFOE
PBRPOL
PCYPOL
QQQBNG01
REKYAI
WANMUU01

Supplementary Table S5. Benzene pyridine analogue CSD codes

AMIPYR
AMPYRD
AMPYRE
HOFGEO
ISNICA
MAGXAV
ZZZHKQ02

Supplementary Table S6. Naphthalene CSD codes

BOTSOT
CUKFOE
HNAPAC
HNAPAC01
JEVMON
JUBBIR
NAPDCX
NAPHOL01
NAPHTA15
NAPMCB
NAPXAC01
NPHHQU10
OGEHOY
PEYGEF
PEYGEF01
QEPGAU
QEXRAN
RACPAM
SAHSID
SAYRIT
SECKES
SOYRAA
VABGIQ
VUPYAH
XEJNUV03
XENLEJ
YODLIN

Supplementary Table S7. Naphthalene pyridine analogue CSD codes

CLQUIN
HXQUIN14
LOCJIX
NONMEI
QEWHOR
SAZNIQ
SECQEY
UPAJIF
WIWCAH
